# War Neuroses and Psychoses

**Published:** 1918

**Authors:** 


### NEUROLOGY

**War Neuroses and Psychoses.** Capt. CLARENCE B. FARRAR. *American Journal of the Medical Sciences,* March 1918.

The author thinks that it may be useful to distinguish the following conditions :

1. Simulated malingering. — This refers to certain pathological mental states with partial or complete irresponsibility.

2. Exaggeration and Perseveration. — Perseveration means the indefinite continuation of symptoms after the original cause has subsided.

In conditions accompanied by pain perseveration may extend over many months, with no discoverable basis.

3. Hysterical Autosuggestion. — In this it is unnecessary to assume any organic lesion whatever underlying the symptoms presented.

4. Mental Contagion. — In its simplest form it amounts to nothing more than purposive collusion or limitation.

Less directly conscious imitation is notoriously effective in the production of neurotic symptoms.

5. Voluntary Fabrication. — These simulated conditions include fictitious skin eruptions, abscesses and ulcus; contusions, erythematous infiltration from self-inflicted blows repeated at intervals; self-inflicted bullet wounds and so forth.

The principal mental diseases encountered in the service are :

Dementia Precox. — Slowly developing psychosis : apathy, inertia, defective sense of personal identity, aural hallucinations purposeless impulsive (often dangerous) acts.

Recognition of the psychosis may be delayed by a practically complete remission of symptoms, which may thus lend weight to the suspicion of malingering.

Manic-depressive Psychosis. — Characterized by recoverable phases of emotional depression with feeling of incompetence and varying degree of inhibition in thought, speech and action on the one hand, and exhilaration, hyperactivity and talkativeness on the other.

Paresis and Taboparesis. — In paresis, brain involvement and mental symptoms are foremost.

Epilepsy and Epileptiform States. — There are other symptoms of epilepsy besides convulsions which may cause trouble : dizzy spells, fits of irritability with violent acts, states of clouded consciousness with amnesia.

The chief epileptiform seizures are :

Genuine epilepsy, post-traumatic epilepsy, hysteria, alcoholism, paresis, arteriosclerosis and brain syphilis, brain tumor, meningitis, and malingering.

Primary Mental Defect. — High degrees of feeblemindedness are easily overlooked by medical boards dealing primarily with physical conditions, but it is well to remember that inattention, forgetfulness, failure to execute orders, erratic and unreliable conduct may sometimes be traceable to this cause.

Psychopathic Constitution. — Constitutional psychopathes are victims of unstable or inharmonious mental development. While judgment defects in particular directions may be conspicuous, they are, in a general way, clear and rational. Once the situation is recognized these men are better eliminated from the service with as little delay as possible.

Neurotic Reactions. — Their primary causes are endogenic.

The neurosis, however, like all purposive conduct, is more specifically a reaction to a given situation. On the other hand, it must not be overlooked that the majority of cases develop in men *constitutionally predisposed.*

Psychogenic factors are generally conceded to be fundamental in the development and perpetuation of war neuroses.

Now it is a striking fact that severe physical trauma and neurosis are, as a rule, mutually exclusive. This applies especially to injuries of brain or cord.

Furthermore, severe neuroses may follow not only concussion from high explosives but also other injuries and diseases, and may develop in men who have never left camp or suffered from any definite illness or accident.

Finally it has been shown that neurotic conditions are relatively more frequent in hospitals distant from the front than in the field hospitals themselves.

Types of War Neuroses. — In the trenches the primordial emotion of fear appears to be universal and physiological, at least as an initial or episodic mental state.

The symptoms may be practically all classified under two headings : neurasthenia and hysteria.

The complaints of neurasthenics generally are multiple, often vague and changeable. At the same time dominant symptoms are so commonly associated with particular organs that we may conveniently speak of the group of *somatoneuroses.*

Somatoneurotic reactions may be : 1) respiratory, 2) cardiac, 3) gastro-intestinal, 4) excretory, 5) musculo-articular.

Hysteria develops, as a rule, abruptly and spectacularly, and oftenest in men young in years and young in service.

Sensory Disorders. — Hysterical blindness and deafness are usually sequent to concussion associated with lesions of the sense organs. Pain reactions of all kinds are usually met with. Cutaneous anesthesia of familiar hysterical types is not uncommon, and often accompanies functional motor disorders. Motor disorders of psychogenic origin include derangement of the speech mechanisms; tics; general trembling, tremor of extremities, choreiform movements; myoclonus; epileptiform convulsions, rhythmic movements, and contractures of all description.

A special type of functional contracture is trench spine.

Psychic Disturbances. — In concussion cases a stuporous state may persist for several days, furnishing the cue for continuing individual symptoms and episodic conditions such as dream-waking, hallucination, hallucination automatism. Loss of memory is a favorite complaint of war neurotics, but cooperation is not always of the sort to make memory tests of value.

Treatment. — It should be started promptly and carried out rapidly and determinedly. There should be as little relaxation of military discipline as possible. For certain shock symptoms, a counter-shock may be all that is required.

A new mental attitude and a new way of self-management should be aimed at to ensure the after condition of the nervous invalid.

Finally it would seem highly desirable to incorporate in the system of training a certain amount of information concerning war neuroses and to teach mental hygiene as well as physical.

